# Clinical Trials of Probiotic Strains in Selected Disease Entities

**DOI:** 10.1155/2020/8854119

**Published:** 2020-05-28

**Authors:** Ruth Dudek-Wicher, Adam Junka, Justyna Paleczny, Marzenna Bartoszewicz

**Affiliations:** Department of Pharmaceutical Microbiology and Parasitology, Faculty of Pharmacy, Medical University of Silesian Piasts, Wrocław, Poland

## Abstract

Probiotics are live microorganisms which when administered in adequate amounts confer a health benefit on the host. Although their mechanism of action is not clearly explained, it is known that they positively modulate the immune system, which leads to immunity potentiation. A number of studies prove that probiotics strengthen cognitive functions, reduce anxiety, and regulate the lipid metabolism in the human body. Probiotics used in humans are most often of the *Lactobacillus* and *Bifidobacterium* species. However, as more research is conducted, new species with beneficial, probiotic properties are being discovered. This paper provides a review of available information about the influence of probiotics on human health. It summarizes the current knowledge on the mechanism of action of probiotics as well as clinical trial results proving their efficacy in allergic, neurodegenerative, and cardiac diseases. This review also discusses the data concerning the safety of probiotics in clinical treatment.

## 1. Introduction

For many years, the development of research has been aimed at characterizing the human intestinal microbiome and determining the role of individual species present in it. Probiotic strains (probiotics) are defined as “live microorganisms which when administered in adequate amounts confer a health benefit on the host” [1]. When ingested or applied to the skin, probiotics interact with the microbiome that inhabits the respective niches of the body. Currently, increased nutritional awareness of consumers in developed societies is observed. The above translates into consumer interest in food products that can not only satisfy hunger but also fulfill additional physiological and nutritional functions, by improving health or preventing diseases. The main advantage of probiotics is their impact on the development of the microbiome in a way that ensures a proper balance between pathogens and bacteria necessary for the proper functioning of the body.

For this reason, probiotics are widely used to restore the normal composition of the microbiome after antibiotic therapy. There are also reports of the special role of probiotics in the prevention and treatment of obesity, diabetes, allergies, asthma, lung diseases, autoimmune diseases, HIV (Human Immunodeficiency Virus) infections, cancers, urogenital infections, and gastrointestinal diseases such as diarrhea, irritable bowel syndrome, necrotizing enterocolitis, or cirrhosis, as well as in the eradication of *Helicobacter pylori* infections. The reader can find these studies in an extensive review by Hill et al. [1].

This article discusses the probable mechanism of action of probiotics and highlights their use in the prevention and treatment of selected disease entities.

## 2. Probiotics

Probiotic properties are associated with specific strains of the microorganisms. In order for a strain to be described as “probiotic,” it has to meet a number of requirements related to safety, functionality, and technological suitability [2, 3]. The safety profile is determined based on the strain's origin, degree of antibiotic resistance, and no relationship to pathogenic strains. When assessing functionality, the ability to survive and maintain metabolic activity and growth at the target site is taken into account, as well as antagonistic activity against pathogens such as *Helicobacter pylori*, *Salmonella* spp., *Listeria monocytogenes*, or *Clostridium difficile*. Technological usefulness is demonstrated, for example, by the effective production of large amounts of biomass and high productivity of cultures and their resistance to bacteriophages [3]. Probiotic microorganisms used in humans mainly belong to the *Lactobacillus* and *Bifidobacterium* species. Not only are they free from lipopolysaccharides that cause inflammation, but they also release active molecules that help keep the intestines and skin healthy. Other commonly used probiotics are *Lactococcus*, *Streptococcus*, and *Enterococcus*, as well as *Bacillus clausii*, *Enterococcus faecium* SF68, and some yeast strains of the genus *Saccharomyces*. The *Escherichia coli Nissle* 1917 strain is a unique probiotic which synthesizes the semirough lipopolysaccharide (LPS) and does not produce P- and S-fimbrial adhesins, which are important virulence factors in other *E. coli* strains. Due to these features, *E. coli Nissle* 1917 has no pathogenic effect and can be used in the treatment of gastrointestinal diseases [4].

A list of the most commonly used probiotic strains contained in pharmaceutical products and used as food additives is presented in Table 1.

## 3. Mechanisms of Action of Probiotics

The mechanism by which probiotics interact with the host is most likely pleiotropic. The strongest clinical evidence of the beneficial effect of probiotics on human health is their immunomodulatory activity manifested in an increase in body immunity [7, 8]. The intestines, together with the gut-associated lymphoid tissue (GALT), play an important role in ensuring and maintaining the body's immunity. Imbalances in this system lead to inflammation in the digestive system. It is believed that increased mucosal permeability and loss of intestinal epithelial cells integrity (IECs) also play a role in the pathogenesis of other diseases [6–8].

It has been shown that probiotics affect every part of the intestinal tissue (such as the mucus barrier, epithelium, lamina propria), vessels and nerves of its components, smooth muscles that control intestinal peristalsis, and mesenteric lymph nodes that have the ability to communicate with the immune system. One of the possible mechanisms of action is a direct improvement of the intestinal defense barrier by reducing mucosal permeability and improving epithelial integrity and the interaction of probiotics with commensal organisms [7]. Probiotics play an important role in regulating both the innate and adaptive immune system by activating macrophages, NK cells, and cytotoxic T cells, by modulating IgA production, stimulating toll-like receptors (TLRs), and modifying the cytokine expression profile [7, 8]. The beneficial effect of commensal bacteria, e.g., lactobacilli, is associated with the stimulation of pattern-recognition receptors (PRRs), especially TLRs, on the surface of enterocytes and dendritic cells. These receptors recognize microbial-associated molecular patterns (MAMPs), and their activation triggers numerous cascades for intracellular transmission, which in turn leads to the production of endogenous antibacterial agents, defensins, proinflammatory cytokines, and chemokines. Thanks to the above, the mechanisms are activated conditioning the tightness of the intestinal barrier and limiting the presence of microorganisms in the intestinal lumen. Through these mechanisms, probiotics facilitate the creation of the immune system of a newborn child, among others, by balancing the activity of helper lymphocyte subpopulations Th1 and Th2 and stimulating regulatory T cell (Treg) subpopulations [9]. It has also been reported that probiotics can produce metabolites with anti-inflammatory local and systemic activity and, as a consequence, indirectly affect the inflammatory reaction. These metabolites are mainly short-chain fatty acids (SCFAs) such as butyric, acetic, and propionic acid [9, 10]. SCFAs bind to G-protein-coupled receptors to induce intracellular signaling. Therefore, SCFAs act as mediators between the microbiome and the brain, thanks to which intestinal bacteria influence the physiology and behavior of the brain [11]. They activate the receptors (FFAR2, FFAR3, or GPR109A) on the intestinal epithelial cells, which leads to an inhibition of nuclear secretion of NF-*κ*B transcription factor by B lymphocytes. SCFAs also have the ability to inhibit histone deacetylases in Treg lymphocytes, which increases their number and consequently suppresses an excessive immune response. SCFAs also stimulate the ileal and colon L cells to produce gastrointestinal hormones such as pancreatic polypeptide YY (PYY) and glucagon-like peptide-1 (GLP-1). In 1993, it was demonstrated for the first time that intravenous administration of the PYY peptide reduced appetite. The main task of GLP-1 is to stimulate insulin secretion, and the receptors for this peptide are found in the digestive system—the intestine and the endocrine part of the pancreas—and in the central nervous system (CNS) [12]. SCFAs stimulate tolerogenic dendritic cells (DCs) that trigger CD4 + T cells to differentiate into Tregs. These effects inhibit the production of cytokines by neutrophils and macrophages by interacting with the receptors. The tryptophan and indole derivatives produced by probiotics interact with the AhR (aryl hydrocarbon receptor) while adenosine and the inosine derivative interact with the adenosine-2A receptor. Both receptors are located on T lymphocytes, and the interactions of the above compounds cause an anti-inflammatory effect. Histamine produced by some probiotic strains interacts with the H2 receptor present on intestinal epithelial cells and macrophages. This interaction results in a decrease in the level of proinflammatory cytokines (TNF-*α*, MCP-1, and IL-12) [10].

Bacteria have the ability to produce and release neurotransmitters such as *γ*-aminobutyric acid (GABA), serotonin, catecholamines, and histamine or their precursors. Neurotransmitters send signals to the CNS through enterochromaffin cells and intestinal nerve receptors. GABA, the main inhibitory neurotransmitter in the CNS, whose dysfunction is associated with depression, anxiety, autism, and schizophrenia, is produced in human intestines by *Lactobacillus brevis* and *Bifidobacterium dentium* [11]. Other products of probiotic metabolism are bacteriocins, which can be compared to an antibiotic. They include acidoline, acidophylline, lactacin, lactocidin, reuterin, lactoline, and enterocin. Still other metabolites exhibit anticancer or immunosuppressive activity. The antimicrobial properties of probiotics include not only the production of antimicrobial compounds, but also competition with pathogens for adhesion to the epithelium and to nutrients. The ability of probiotic strains to coaggregate enables the formation of a protective barrier preventing the colonization of the epithelium by pathogens. In addition, they have the ability to inhibit the production of bacterial toxins. Probiotics have been proven to increase the synthesis and absorption of vitamins (mainly from group B, but also PP and K) and mineral compounds and to stimulate the production of organic acids and amino acids. They may also be able to produce mucus as well as enzymes such as esterase, lipase, and coenzymes A, Q, NAD, and NADP [2, 13]. Probiotics have been shown to effectively lower total cholesterol and low-density lipoproteins (LDL). There are several proposed mechanisms of action of probiotics on total cholesterol and LDL levels. These are enzymatic deconjugation of bile acids by hydrolysis of bile salts, ability to bind cholesterol in the small intestine, assimilation and incorporation of cholesterol into cell membranes of probiotics, conversion of cholesterol into coprostanol, or reduction of cholesterol esters in LDL particles [13].

It can also be assumed that probiotic strains may be responsible for the detection and degradation of potential carcinogens [13, 14]. Lactic acid bacteria present in the intestine play a role in carcinogenesis regression because of their effect on immunomodulation. In addition, they have the ability to both increase and decrease the production of anti-inflammatory cytokines, which play an important role in preventing carcinogenicity. They are also able to activate phagocytes to eliminate cancer cells at an early stage. The use of probiotic bacteria killed with high temperature in combination with radiation helped increase the detection of cancer cells. A reduced resistance to carcinogens has been observed in germ-free mice. There are many cohort studies showing a correlation between the consumption of dairy products and the risk of colon and colorectal cancer [14].

## 4. Allergic Diseases

Allergic diseases have become a serious health concern in recent decades. The number of cases of atopic dermatitis, food allergies, or asthma is constantly increasing, especially in Western societies. To explain this phenomenon, the “hygiene hypothesis” is often invoked, which claims that a reduced exposure to microbes in early life leads to imbalances between Th1/Th2 lymphocytes. This hypothesis is confirmed by studies of the GABRIEL group (“A Multidisciplinary Study to Identify the Genetic and Environmental Causes of Asthma in the European Community,” GABRIEL, Advanced Study), which clearly indicate that the risk of atopy or asthma was significantly lower in children who had contact with environmental microorganisms compared to children raised in cities [15]. Numerous studies were conducted to explain the complicated mechanism of allergy development [16–23]. In a clinical trial it was observed that *L. rhamnosus* GG (LGG) reduces the concentration of exhaled nitric oxide in 4–7 year olds with asthma. However, the use of *Lactobacillus reuteri*, *Lactobacillus rhamnosus* HN001, *Lactobacillus paracasei* subsp. *paracasei* F19, *Bifidobacterium bifidum, B. lactis,* and *Lactococcus lactis* in infants did not reduce asthma symptoms [17]. More favorable results were obtained by examining the effectiveness of probiotics in the prevention and treatment of eczema and atopic dermatitis. Kukkonen et al. proved that giving pregnant women complex probiotics (*Lactobacillus rhamnosus, Bifidobacterium breve,* and *Propionibacterium freudenreichii*) significantly reduces the risk of atopic dermatitis in children up to the age of two [18]. The preventive effect of LGG has also been demonstrated in another clinical study conducted on pregnant women in New Zealand [19]. A meta-analysis of 21 clinical trials in which prenatal and postnatal women were given probiotics showed that they are effective in preventing but not treating atopic dermatitis [20]. The results of another meta-analysis showed the preventive effect of probiotics on the development of eczema, but the effectiveness of the use of probiotics for other allergic diseases has not been confirmed. However, the effect is moderate, and the only probiotic strain with reproducible data is *Lactobacillus rhamnosus* GG (LGG). The World Allergy Organization (WAO) suggests the use of probiotics in pregnant and lactating women and infants only if there is a high risk of hereditary allergies [21].

The dramatic increase in the prevalence and severity of food allergies around the world is also prompting the search for new therapeutic strategies. Dysbiosis of intestinal microbiome early in life is a critical factor in the development of food allergies. The intestinal microbiome is therefore a promising goal of innovative therapeutic and prophylactic strategies. Evidence confirms that probiotics modulate immune tolerance by affecting the structure and function of intestinal microbiota, interacting with enterocytes, and changing nonimmune mechanisms such as intestinal permeability and mucus thickness and through immunogenic mechanisms including the stimulation of sIgA and *β*-defensin production, as well as modulation of cytokine response by immune cells. A beneficial effect in alleviating food allergies has been proven for *L. plantarum* and *B. adolescentis* and for the probiotic mixture *L. casei* W56*, L. lactis* W58, *L. acidophilus* W55, *L. salivarius* W57, *B. infantis* W52, *B. lactis* W18, and *B. longum* W51 [22]. Neau et al. confirmed the preventive effect on food allergy to cow's milk of 3 new strains of *Lactobacillus salivarius* LA307, *Bifidobacterium longum* subsp. *infantis* LA308, and *Lactobacillus rhamnosus* LA305 [23]. In a clinical study conducted on 220 children with confirmed allergy to cow's milk, it was shown that the administration of highly hydrolyzed casein in combination with the strain *Lactobacillus rhamnosus* GG reduced the occurrence of allergic symptoms and accelerated the acquisition of cow's milk protein tolerance [24].

Peanut allergy is the second most common allergic condition, especially in children. Australian researchers conducted a study that used probiotic and peanut oral immunotherapy (PPOIT). The therapy lasted for 18 months, and *L. rhamnosus* CGMCC1.3724 was used as a probiotic. At the end of the study, it was observed that 89.7% of the participants in the PPOIT group were desensitized compared to 7.1% in the placebo group. In addition, the researchers checked the condition of the children participating in the study after 4 years. Their allergic reactions to nuts were checked, skin tests were performed, and the concentration of sIgE and sIgG4 antibodies to peanuts was measured. It was shown that 67% of children from the PPOIT group could safely eat peanuts. Although the results of the study are promising, further research is needed to confirm that PPOIT therapy may become a cure for peanut allergy [25, 26].

The results of the above-described studies indicate the high therapeutic potential of probiotics, although further, more detailed research is necessary to fully understand the potential of probiotics in the fight against allergic diseases.

## 5. Heart and Circulatory System

Cardiovascular disease (CVD) is a leading cause of death worldwide. There are numerous reports on the beneficial properties of certain probiotic strains used in treatments aimed at lowering cholesterol and treating hypertension. It has been proven that probiotics directly protect against strokes, by producing certain proteins and by activating heat shock proteins, and also that they prevent hypertrophy after a heart attack. In addition, they regulate fat metabolism, affecting the size of fat cells, and they regulate the circulation of leptin and adiponectin. It has also been found that proteins produced by LGG such as p75 have dose-dependent cardioprotective effects and reduce the risk of ischemic stroke. Similar properties were observed for proteins produced by *L. plantarum* 299v [27]. The cardioprotective effect of probiotics on the heart muscle has been proven in animal model studies. Inhibition of proinflammatory cytokine production and reduction of oxidative stress have also been exhibited by *B. breve, L. casei, L. bulgaricus*, and *L. acidophilus*. Therefore, probiotic supplements may find use as an additional prophylactic option in patients at risk of coronary heart disease [28, 29]. High cholesterol, especially the LDL fraction, is a major precursor of hypertension, hyperlipidemia, and coronary heart disease and also causes plaque buildup in the arteries. The serum LDL fraction maintained within the optimal range reduces the chances of these diseases occurring. In a study in which a meta-analysis of randomized clinical trials was conducted on 1971 patients, it was shown that probiotic strains, i.e., *L. acidophilus, L. Lactis,* and *L. plantarum,* significantly reduce the level of total serum cholesterol [30]. Lowering the total cholesterol and LDL fraction in all groups compared to the control group was observed in a study involving 485 patients with high, borderline, and normal cholesterol [31]. In clinical trials, strains such as *Lactobacillus plantarum* ECGC 13110402, *Lactobacillus fermentum* ME-3, *Bifidobacterium lactis* HN019, *Streptococcus thermophilus*, *Lactobacillus acidophilus* L1, *Bifidobacterium longum* BL1, and *Lactobacillus plantarum* 299v showed a total cholesterol level lowering effect. The most favorable results were obtained in the case of *L. acidophilus* and *L. plantarum* [13]. To date, Health Canada has only approved one probiotic product recommended for the treatment of cardiovascular disease. The product-Cardioviva™, also available in the USA and Europe, contains 2 billion encapsulated *Lactobacillus reuteri* NCIMB 30242 bacteria, which have been clinically proven to lower LDL cholesterol by 11.6% in adults with hypercholesterolemia [32].

Hypertension is closely related to hypercholesterolemia. It has been proven that selected strains of the genera *Lactobacillus* and *Bifidobacterium* are effective in lowering blood pressure because they produce peptides that act similarly to drugs from the group of angiotensin converting enzyme (ACE) inhibitors [33]. A meta-analysis of 14 clinical trials with 702 participants showed that fermented probiotic milk significantly reduced both systolic and diastolic blood pressure in hypertensive patients. The increase in the population of *Lactobacillus* and *Bifidobacterium* caused by the administration of fermented milk caused a proportional increase in proteolytic activity and inhibition of ACE [34].

The available meta-analyses have some limitations, and hence more randomized multicenter studies should be conducted to broaden the knowledge about the possibility of using probiotics in the treatment of cardiovascular diseases.

## 6. Neurodegenerative Diseases

Numerous studies describe the effect of intestinal microbiome on human health and homeostasis. Moreover, research also describes the possible role of the microbiome in the pathogenesis of brain function disorders. Neuropsychiatric diseases have various causes. The emerging evidence of the interaction between the brain, intestines, and microbiome can help explain the mechanisms underlying these complex interactions. A two-way information exchange takes place on the gut-brain axis. Direct and indirect exchange mechanisms include nerve (vagus, intestinal nerves), hormonal (serotonin, monoamines, GABA, neutrophilic brain factor), and immune pathways. It is believed that changes in the intestinal microbiome are a possible cause of some brain diseases, including Parkinson's disease (PD), Alzheimer's disease (AD), and multiple sclerosis (MS). In studies conducted on adult patients, a reduction in the percentage of *Prevotellaceae* was found in the stool samples of patients with PD compared to the control group. A greater share of *Enterobacteriaceae* was observed in people with postural instability and movement difficulties. The composition of the microbiome was also studied in children with relapsing-remitting MS. A reduction in the number of *Fusobacteria* was significantly associated with the risk of recurrence. A decrease in the number of bacteria from the *Prevotella* and *Lactobacillus* genera has been observed in people with MS [10]. In the light of the above findings, it seems reasonable to test probiotics for their properties supporting the treatment of PD, AD, and MS diseases. *α*-Synuclein plays an important role in the process of neurodegeneration. It is a protein abundant in neurons, whose physiological role is not fully understood. However, a wealth of data indicates its involvement in the release of neurotransmitters from synaptic endings and in neuronal plasticity. Normal functions of this protein are disturbed during its aggregation. Aggregated *α*-synuclein is involved in cell death in neurodegenerative diseases, including Parkinson's disease, Alzheimer's disease with Lewy bodies, and dementia with Lewy bodies. *α*-Synuclein activates TLR receptors, which leads to the formation of an inflammatory reaction preceding the degeneration of neurons. Therefore, inhibition of TLR receptors delays the progression of PD or AD. The best known probiotic strains of the genera *Lactobacillus* and *Bifidobacterium* interact with TLRs, helping to reduce the inflammation. Therefore, they can be important in preventing the degeneration of neurons [35]. A study by Akbari et al. showed that a probiotic supplement consisting of *Lactobacillus acidophilus*, *Lactobacillus casei*, *Bifidobacterium bifidum*, and *Lactobacillus fermentum* administered for 3 months improves the cognitive functions of patients with AD [36]. The effectiveness of “psychobiotics” has also been confirmed in a study conducted on 20 patients with Alzheimer's disease. Markers of intestinal inflammation were examined and stool samples were analyzed before and after 4 weeks of supplementation with a probiotic consisting of *Lactobacillus casei* W56, *Lactococcus lactis* W19, *Lactobacillus acidophilus* W22, *Bifidobacterium lactis* W52, *Lactobacillus paracasei* W20, *Lactobacillus plantarum* B62, *Lactobacillus plantarum* W23, and *Lactobacillus salivarius* W24. After treatment, an increase in *Faecalibacterium prausnitzii* was observed compared to baseline. At the same time, kynurenine serum level has increased. The results indicate that, in patients with Alzheimer's disease, supplementation with a multispecies probiotic affects the composition of intestinal bacteria, suggesting that microbiome modulation may affect the development and course of Alzheimer's disease [37]. However, different results were obtained in a study of 60 patients aged 65–90 years in a very advanced stage of Alzheimer's disease. They received mixtures of probiotic strains: either *Lactobacillus fermentum, Lactobacillus plantarum,* and *Bifidobacterium lactis* or *Lactobacillus acidophilus, Bifidobacterium bifidum,* and *Bifidobacterium longum* for 3 months. This study showed that patients were insensitive to treatment. The authors emphasize that negative test results may be due to the use of other bacterial cultures and the inclusion of patients at a very advanced stage of the disease [38].

In a clinical trial, Parkinson's patients were given a mixture of the following strains for 12 weeks: *Lactobacillus acidophilus* (2 × 109 CFU/g), *Lactobacillus reuteri* (2 × 109 CFU/g), *Bifidobacterium bifidum* (2 × 109 CFU/g), and *Lactobacillus fermentum* (2 × 109 CFU/g). Improved movement and parameters such as CRP level, glutathione concentration, and insulin sensitivity were observed in the examined group [39]. Another study showed that oral administration of the *Lactobacillus casei Shirota (LcS)* strain for 4 weeks improved the motor function and reduced spasticity in the limbs. Furthermore, administration of the same strain led to a significant reduction in anxiety symptoms compared to the control group in patients with chronic fatigue syndrome. In addition, the administration of the same strain reduced abdominal pain, reduced flatulence, and improved stool consistency in PD patients. A beneficial effect, relieving the symptoms of Parkinson's disease, was also observed after the administration of *L. helveticus* R0052, *B. longum* R0175, *B. animalis* subsp. *lactis*, *S. thermophiles, L. bulgaricus*, and *L. lactis* subsp. *lactis* [40, 41]. One of the latest review articles discusses microbiological therapy as a novel treatment for Parkinson's disease. This analysis also contains data on the effects of stool transplantation in patients with Parkinson's disease [42].

It has been observed that MS patients have increased gene expression associated with proinflammatory immune pathways, including interferon, TLR, IL-6, and dendritic cell maturation. In these patients, a larger population of the *Akkermansia* and *Methanobrevibacter* species was also noted, compared to the number of these bacteria in the healthy control group. *Akkermansia* is a mucus-degrading bacterium, which in turn disturbs the functioning of the intestinal barrier, so there may be a direct relationship between the composition of the microbiota and the occurrence of MS [43]. Patients with MS also have a reduced amount of *Lactobacillus* bacteria. The administration of these probiotic strains resulted in increased bacterial diversity in the microbiome. Thus, the use of probiotics reduced the dysbiosis characteristic of MS and reduced the number of bacteria of the genera *Akkermansia* and *Blautia*. At the immune level, the administration of probiotics induced an anti-inflammatory peripheral immune response. In addition, in the control group with the MS risk gene (HLA-DQA1), a reduced expression of this gene was observed. These results suggest that probiotic treatment may have a synergistic effect [44]. There is also a study suggesting that *L. reuteri* alleviates the symptoms and improves the quality of life of patients with multiple sclerosis [10].

The potential usefulness of probiotics in the prevention or treatment of neurodegenerative diseases is becoming a widely studied topic. However, further research is needed to optimize the composition and dosage of simple and complex formulations and to develop optimal therapeutic regimens for individual neurodegenerative diseases. In addition, future research should also take into account the tolerability and safety of probiotics in patients with neurodegenerative diseases, as well as hormonal, immunological, neurochemical, and metabolic changes induced by probiotics or prebiotics. Therefore, further investment in large-scale clinical trials is needed to prove the efficacy of probiotics in neurodegenerative diseases [45].

## 7. Probiotics Safety

The American Food and Drug Administration (FDA) describes probiotics as Generally Recognized as Safe (GRAS). Based on the analysis of randomized clinical trials, potential applications of probiotics include the treatment of cystic fibrosis (e.g., *Lactobacillus* GG), autism spectrum disorders (e.g., *L. plantarum* WCFS1), Alzheimer's disease (e.g*., L. acidophilus, L. casei, B. bifidum, L. fermentum*), and celiac disease (e.g., *B. infantis* NLS, *B. longum* CECT 7347, *B. breve* BR03, *B. breve* B632); prevention of caries (e.g., *Lactobacillus GG*), pneumonia associated with mechanical ventilation (e.g., *L. casei rhamnosus, L. plantarum* 299, *B. longum* + *L. bulgaricus* + *Str. thermophilus*), and urinary tract infections (e.g., *Lactobacillus* GG); and prevention of radiation-related symptoms (e.g., *L. casei* DN-114001). However, there are situations in which probiotics should not be administered. Such situations include immunosuppressive treatment, anticancer treatment, and acute pancreatitis. Caution is advised in the use of probiotics in premature babies, in patients with immunodeficiency, in patients with a catheter inserted into large veins, or in patients with a severe clinical condition. The administration of probiotics by jejunostomy may also be a risk factor. Cases of sepsis have also been reported in children with short bowel syndrome receiving LGG supplementation. In addition, several dozen cases of fungemia have been reported in individuals receiving *S. boulardii* as well as bacteremia in individuals receiving bacterial probiotics. In rare cases, probiotic bacteria may contain antibiotic resistance genes that they can pass on to other strains of bacteria, including harmful strains that cause infections [46, 47]. Although probiotics are widely used in the treatment of postantibiotic diarrhea and *Clostridium difficile* infections, there is a lack of unambiguous data confirming the legitimacy of such therapy [48, 49].

## 8. Summary

Knowledge about the human intestinal ecosystem and its role in the maintenance of health is still limited. The mechanism of action of probiotic strains remains to be fully explained. Modulation of the microbiome composition and the effect of probiotics on the human body occurs through mechanisms such as bacteriocin and SCFA production, epithelial cell barrier modulation, innate immunity modulation (dendritic cell maturation), effect on Th1 to Th2 ratio, increase in the number and activity of regulatory T cells, and degradation of carcinogenic compounds [9–14].

A thorough understanding of these mechanisms is necessary to make full and safe use of the potential benefits that probiotic strains may bring to patients suffering from allergic, neurodegenerative, or cardiological diseases. The effectiveness of probiotics may vary depending on treatment, disease, and strain or strains that make up the probiotic. In addition, a probiotic may be effective in treating the disease, but not in its prevention. Research to date suggests that probiotics have beneficial and multifaceted effects on human health, which encourages further experimental and clinical research. Some probiotic activities are well documented, and their use alone or in combination with other therapies can be considered “evidence-based.” However, in many cases further research is needed because the available evidence is insufficient to demonstrate the effectiveness of the probiotics themselves. Carefully designed multicenter clinical trials are needed to confirm the performance of individual probiotic strains administered at specific doses and to clarify the duration of therapy. Considering the contraindications to the use of probiotics and possible side effects, further research is needed on their safety.

## Figures and Tables

**Table 1:** Exemplary probiotic strains used in humans [2, 5, 6].

Genera *Lactobacillus*	Genera *Bifidobacterium*	Genera *Streptococcus*	Other
*L. acidophilus* *L. amylovorus* *L. casei* *L. delbrueckii* *L. fermentum* *L. gasseri* *L. helveticus* *L. johnsonii* *L. pentosus* *L. plantarum* *L. reuteri* *L. rhamnosus*	*B. adolescentis* *B. animalis* *B. bifidum* *B. breve* *B. infantis* *B. longum*	*S. thermophilus* *S. lactis* *S. intermedius* *S. salivarius* *S. cremoris*	*Saccharomyces boulardii* *Escherichia coli Nissle 1917* *Enterococcus faecium SF68* *Bacillus clausii* *Lactococcus lactis* *Propionibacterium freudenreichii* *Pediococcus acidilactici*

## Data Availability

The data supporting the conclusions of this paper are available through the articles cited in the reference list.
