# Corrigendum to “Clinical Trials of Probiotic Strains in Selected Disease Entities”

**DOI:** 10.1155/2021/7356890

**Published:** 2021-02-11

**Authors:** Ruth Dudek-Wicher, Adam Junka, Justyna Paleczny, Marzenna Bartoszewicz

**Affiliations:** Department of Pharmaceutical Microbiology and Parasitology, Faculty of Pharmacy, Medical University of Silesian Piasts, Wrocław, Poland

In the article titled “Clinical Trials of Probiotic Strains in Selected Disease Entities” [1], the funding information in the Acknowledgments section should be corrected as follows:

This publication was prepared under the project financed from the funds granted by the Ministry of Science and Higher Education in the “Regional Initiative of Excellence” Programme for the years 2019–2022, project number 016/RID/2018/19, and the amount of funding is 11,998,121.30 PLN.
